# Diluting a diet with oat hulls of different particle size enhances gastrointestinal development and function of weanling pigs

**DOI:** 10.1093/jas/skaf377

**Published:** 2025-10-25

**Authors:** Tetske G Hulshof, Hubèrt M J Van Hees, Geert P J Janssens, Michael O Wellington

**Affiliations:** Swine Research Centre, Trouw Nutrition R&D, Boxmeer, 5831 JN, The Netherlands; Swine Research Centre, Trouw Nutrition R&D, Boxmeer, 5831 JN, The Netherlands; Department of Veterinary and Biosciences, Ghent University, Merelbeke, 9820, Belgium; Swine Research Centre, Trouw Nutrition R&D, Boxmeer, 5831 JN, The Netherlands

**Keywords:** gastrointestinal development, insoluble dietary fiber, oat hulls, stomach functioning, weanling pig

## Abstract

Insoluble dietary fibers (IDF), especially in coarse form, are considered beneficial for gastrointestinal tract (GIT) development in weanling piglets. This study aimed to determine the effect of feeding diets with high IDF by including 15% oat hulls (OH), either finely or coarsely ground, on GIT development in weanling piglets. A total of 72 piglets (Hypor Libra × Hypor Maxter, 26 ± 0.6 d, 7.0 ± 1.2 kg bodyweight [BW]) were housed in three large pens (24 piglets/pen). Each pen was equipped with three electronic feeding stations and each station contained one of three experimental diets (*n* = 8 piglets/dietary treatment). The control (CON) diet consisted of finely ground highly digestible ingredients. For the OH diets, 15% finely ground (OH-f) or 15% coarsely ground OH (OH-c) were included at the expense of corn starch. A total of 12 randomly selected piglets/diet were euthanized on d13 post-wean for GIT measurements. Empty stomach weight was highest for OH-c, followed by OH-f, and CON (9.66, 8.78, and 7.32 g/kg BW, respectively; *P* < 0.001). Weights of gastric content (*P* = 0.028), cecum content (*P* = 0.032), empty colon (*P* = 0.027), and colon content (*P* = 0.024) were higher for OH-f and OH-c compared to CON. Piglets fed the OH-c diet had a higher pH gradient (0.93; *P* < 0.001) and smaller DM gradient (−2.3%-point; *P* = 0.025) between the proximal and distal area of the stomach compared to both CON (−0.12 and −9.4%-point, respectively) and OH-f (0.23 and −4.3%-point, respectively) caused by higher pH (*P* = 0.004) and DM (*P* < 0.001) in the proximal stomach. Total VFA concentrations were higher in OH than for CON in the stomach, cecum and colon especially for OH-c. Although not significant, the goblet cell counts and goblet cell area were numerically higher in both OH diets than in CON (+30% and +45%, respectively). In conclusion, diluting a nutrient-dense diet by inclusion of 15% OH in the immediate post-weaning period resulted in a more developed and functional GIT (i.e., increased goblet cell counts and area, increased organs weights and increased total VFA production), which was even more pronounced when the OH were in a coarse form.

## Teaser

The inclusion of oat hulls fiber in a coarse form in the diet of weanling piglets may be an effective strategy to improve intestinal development and function.

## Introduction

Feral piglets are observed to consume non-milk objects, including earth and plant material already at a young age (Jensen et al., 1991; Van Hees et al., 2023a). In contrast to feral piglets, commercially kept piglets are fed diets formulated to contain highly digestible and palatable ingredients around the time of weaning (i.e., approximately 28 d of age). The inclusion of fibrous ingredients is commonly avoided in commercial diets. In a recent study, mimicking the feral piglet diet, it was reported that providing chopped grass hay next to a commercial creep feed to suckling piglets increased the weight and length of the gastrointestinal tract (GIT), increased villus height in jejunum and lowered crypt depth in mid-colon, and increased acetic acid, propionic acid and total volatile fatty acid (VFA) production in cecum (Yao et al., 2023). This observation indicates that feeding fiber-rich diets to suckling piglets could be a strategy to kickstart the GIT development process. Recently, similar results were observed in a study where suckling piglets were fed diets with 15% oat hulls (OH) resulting in improved GIT morphology and changes in microbial profile in the colon (Van Hees et al., 2023b).

Development of the GIT is not a process limited to the suckling phase only, but a relevant process during the early post-weaning period as well. It is generally thought that diets containing high-fibrous ingredients, such as OH, support GIT development and health in the post-weaning pig (Bach Knudsen et al., 2012). For example, Yu et al. (2016), showed that a high-fiber diet (14.6% neutral detergent fiber [NDF]) resulted in increased fecal microbiota content in weaned piglets which shows that, also in these young animals, fiber promotes the proliferation of intestinal microbiota. Furthermore, feeding a diet high in insoluble dietary fiber (IDF; by adding barley hulls) to nursery piglets for 9 d, resulted in improved gut morphology, that is, higher ileal villi length, and mucosal enzyme activity (Hedemann et al., 2006). These studies demonstrate that when high-fiber diets are introduced in the early post-weaning period, GIT development can be improved, although effects on growth performance remain inconsistent. The inconsistencies in the growth performance data may partly be addressed by the particle size of these diets. In commercial swine diets, it is considered that fine grinding and pelleting improves feed efficiency through improved digestibility but in general GIT health is compromised (Vukmirović et al., 2017).

In a recent study, combining coarsely ground cereals (i.e., wheat, barley, and wheat bran) with finely ground soybean meal in a pelleted diet resulted in the best growth performance and lowest diarrhea incidence in nursery piglets challenged with F4-positive enterotoxigenic *Escherichia coli* (Hulshof et al., 2024). Moreover, using finely ground ingredients affected gastric and colonic structure, such as reducing mucous-secreting cells and crypt depth (Brunsgaard, 1998; Grosse Liesner et al., 2009). It appears that dietary fiber (DF) loses part of its health promoting properties when ground fine compared to coarse (Millet et al., 2010; Molist et al., 2012) but coarse grinding of DF was found to reduce the palatability of nursery piglets fed diets containing 13% inclusion of wheat bran, sugar beet pulp, alfalfa meal, or carob meal (Solà-Oriol et al., 2009). Thus, optimizing the interaction between high DF diets and processing technologies, such as grinding and pelleting, are considered beneficial for both GIT development as well as feed efficiency, but further studies are warranted.

Oat hulls are a rich source of IDF due to its high cellulose and lignin content (Bach Knudsen, 1997). The addition of 2% OH to an extruded rice-based diet reduced the incidence of post-weaning diarrhea during the first 14 d after weaning and also reduced the apparent total tract digestibility (ATTD) of dry matter (DM), crude protein (CP), and gross energy (GE) but without affecting growth performance (Kim et al., 2008). Addition of 2 or 4% cooked OH to a rice-based diet increased feed intake of pigs in the first 3 wk after weaning while this was reduced when added to a maize-based diet (Mateos et al., 2006). In the same study, OH tended to reduce the incidence of diarrhea irrespective of diet, and, here, OH did not affect ATTD of nutrients (Mateos et al., 2006). Recently in suckling piglets, we have observed that supplemental diets containing micronized cellulose fiber or (fine and coarse) OH stimulated GIT growth and modified hindgut microbial activity resulting in higher VFA concentration (Van Hees et al., 2019; Van Hees et al., 2023b), supporting other studies (Correa-Matos et al., 2003; Zhang et al., 2016; Mu et al., 2017). Thus, the impact of (coarse) high DF diets on feed intake and GIT development (e.g., GIT morphology and function) has been demonstrated in the pre-weaning phase. Pre-wean feed intake is often low and not all piglets in a litter consume creep feed. It is, therefore, relevant to determine the impact of (coarse) high DF diets on GIT development in the early post-weaning piglet, since GIT development and health challenges remain.

Therefore, the goal of the present study was to investigate the effects of feeding diets with high IDF by including 15% OH, either finely or coarsely ground on GIT development and function in early-post weaned piglets. We hypothesized that diets containing OH especially when coarsely ground, would increase goblet cell number and size, increase VFA production and stomach compartmentalization for optimal gastrointestinal development.

## Materials and Methods

### Animal ethics

The experiment was approved by the Dutch Central Authority for Scientific Procedures on Animals (CCD, The Hague, The Netherlands) with animal use protocol AVD2040020184665 and was performed at the Trouw Nutrition R&D Swine Research Centre (Sint Anthonis, The Netherlands).

### Animals, housing, and experimental design

The piglets used in the present study were selected from 10 litters (Hypor Libra × Hypor Maxter; Hendrix Genetics, Boxmeer, The Netherlands) and processed following routine management procedures, including ear tagging, tail docking, and an iron injection within 3 d after birth. Teeth were not clipped. The pre-weaning protocol was followed as described in Van Hees et al. (2023b). Briefly, two weeks before weaning, piglets were offered either a control (CON) nutrient-dense (12% total DF) diet or a high DF diet (15% OH; 20% total DF) containing either fine (OH-f) or coarse (OH-c) OH. A total of 72 piglets that had been offered these diets pre-weaning, were weaned at an average age of 26 d (± 0.6 SD) and 7.0 kg bodyweight (BW) (± 1.2 SD) and continued receiving the same diet for a 13-d period post-weaning. The selected piglets consumed on average 42% less (on average 10 g DM per day per piglet) of these diets during the suckling phase compared to the piglets selected as high creep feed eaters as reported in Van Hees et al. (2023b) and, therefore, pre-wean intake was considered to have a low impact. Post-weaning, the piglets were housed in a temperature-controlled room, set at 30 °C at weaning which gradually reduced to 25 °C by the end of the study. The light system applied 16 h of light and 8 h of darkness. The room contained three large pens (3.0 m × 5.0 m) with three electronic feeding stations (EFS, Schauer Agrotronic, GmbH, Austria) per pen. There were 24 piglets assigned to a large pen based on pre-wean dietary treatment and weaning weight. Each EFS carried one of three experimental diets and eight piglets within each pen could only access their assigned experimental diet (*n* = 8 pigs/EFS) which was the same as received pre-weaning.

Overall, there were 24 pigs/treatment, with each pig used as the experimental unit. The EFS was activated by the pig’s ear tag Radio-Frequency IDentification (MS Tag Round HDX STF-YELOPPRPB1, MS Schippers BV, Hapert, The Netherlands) enabling individual monitoring of feed intake. The EFS had 15 cm feeder space, and one pig at a time could access the feeder. The experimental diets were pelleted at low temperatures (max 62 °C) in a 5 × 35 mm die and then crumbled (35 mm gap setting). The crumbles were fed as-is after weaning. Piglets had ad libitum access to water through nipple drinkers. All diets were formulated to meet or exceed the nutrient requirements of this category of piglets (NRC, 2012) and did not contain antibiotics or therapeutic levels of zinc. The control diet consisted of finely ground highly digestible ingredients (Table 1). The test diets were formulated with 15% OH, either OH-f or OH-c replacing extruded corn starch (Table 1). To the diets, TiO_2_ was added at 0.3% in the formulation from d5 post-weaning onwards to allow digestibility calculations. The OH were acquired from a commercial cereal mill and contained 8% moisture, 1.6% soluble dietary fiber (SDF) and 73.1% IDF (Van Hees et al., 2023b). Half of the OH was coarsely ground over a 3 mm sieve. To obtain finely ground OH, the hulls were milled two times over beater cross mill (Peppink Mills BV, Olst, The Netherlands), with 3- and 1.5-mm sieves and subsequently a 500 µm sieve to remove any remaining large particles (HeMe, Waddinxveen, The Netherlands).

**Table 1.** Ingredient and nutrient composition and wet sieving analysis of the experimental diets fed from weaning up to the day of post-mortem examination

Item	CON	OH-f	OH-c
**Composition^1^, g/kg as-fed basis**			
Wheat	251	251	251
Barley	247	247	247
Corn starch, heat-treated	150		
Oat hulls, finely ground		150	
Oat hulls, coarsely ground			150
Soybean products	96	96	96
Extruded oats	60	60	60
Dairy whey products	49	49	49
Vitamins and minerals	28	28	28
Fats and oils	29	29	29
Potato protein	20	20	20
Sugar	20	20	20
Wheat gluten	20	20	20
Synthetic amino acids	17	17	17
Organic acids as preservatives	7.8	7.8	7.8
Salt	4.7	4.7	4.7
Phytase	0.3	0.3	0.3
**Calculated nutrients^2^, g/kg as-fed**			
Lactose	50.0	50.0	50.0
Ca	6.01	6.25	6.25
P	5.82	5.64	5.64
Digestible P	4.16	4.13	4.13
6-Phytase (FTU)	366	366	366
Na	3.00	3.02	3.02
K	6.04	6.12	6.12
Cl	5.62	5.67	5.67
NE (MJ)^2^	10.38	9.39	9.39
SID Lys	12.49	12.33	12.33
SID Met+Cys	7.60	7.27	7.27
SID Thr	8.31	8.04	8.04
SID Val	8.85	8.47	8.47
SID Trp	2.48	2.44	2.44
**Nutrients, g/kg as-fed basis**			
DM	896	902	904
OM	851	852	852
CP	168	166	168
Crude fat	45	47	47
Starch	415	333	327
Total dietary fiber	120	199	218
Soluble dietary fiber	18	21	23
Insoluble dietary fiber	102	178	195
Wet sieving analysis, % fraction			
3500 µm	0.3	0.4	1.5
2000–3500 µm	0.6	0.6	1.9
1600–2000 µm	1.1	0.8	1.5
1000–1600 µm	2.9	2.7	4.0
400–1000 µm	14.3	13.1	14.3
200–400 µm	10.4	11.2	7.3
< 200 µm	70.4	71.2	69.5

CON = low fiber control diet; OH-f and OH-c contained finely or coarsely ground oat hulls, respectively; FTU = phytase units; SID = standardized ileal digestible.

1 The diets supplied per kilogram feed: 9169 IU vit A, 2002 IU vit D3, 150 IU vit E-acetate, 1.5 mg menadione, 1.6 mg thiamine mononitrate, 4.3 mg riboflavin, 1.9 mg pyridoxine, 30.0 µg cyanocobalamine, 22.4 mg niacin, 12.0 mg calcium D-pantothenate, 683 µg folic acid, 43 µg biotin, 113.7 mg choline chloride, 50.1 mg betaine, 216.8 mg iron, 1.1 mg iodine, 144.2 mg copper, 52.0 mg manganese, 125.6 mg zinc, 0.35 mg sodium selenite.

2 Based on Trouw Nutrition’s internal nutritional database.

3 NRC (2012).

### Euthanasia and post-mortem examinations

A total of 12 piglets/treatment with 4 piglets/EFS were randomly selected from the total of 24 piglets per treatment, for euthanasia on d 13. Thus, a total of 36 piglets were sacrificed on d 13 to examine GIT development and function. Piglets were euthanized by an intracardial injection with 40% barbiturate pentobarbital and immediately exsanguinated. The abdominal organs were removed, and the empty BW was determined. The GIT sections were identified, and fissures were used to retain the intestinal contents. The stomach was first separated from the rest of the GIT, its full weight was measured, a pH probe was inserted in the fundic and pyloric part to measure the pH in each section, and, subsequently, the contents of the fundic and pyloric area were collected separately in a 50 mL tube.

The full weights of the cecum and colon were measured after removing the mesentery ligaments and the empty weights were measured after removing the contents. The contents of the stomach, ileum, cecum, colon, and rectum were collected and stored at −20 °C for later chemical analyses. A subsample of the stomach, cecum, and colon content was collected in tubes containing 50% sulfuric acid, for the analysis of lactate, VFA, and ammonia-N. The length of the colon was determined without stretching. Tissue samples from the gastric pyloric sphincter (whole sphincter), ileocecal valve (3 cm), and cecum (2 cm × 2 cm) were collected in a 4% formaldehyde solution for later microscopic examination. The *pars non-glandularis* was examined for hyper-keratinization (score ranging from 0 [intact mucosa] to 5 [hyperkeratosis and large erosions]).

### Microscopic examination of GIT sections

A longitudinal section of the gastric pyloric region was made per piglet after embedding the tissue in 4% formaldehyde. Each section had a thickness of 5 µm and was embedded in paraffin. The sections of the gastric pyloric region were made visible by using hematoxylin and eosin staining and were made following a standardized protocol. The longitudinal sections were analyzed using a microscope (Nikon Eclipse Ni) with a magnification of 40×. Image material of the sections was collected using a camera (Nikon DS-Fi3) and the material was stitched with the help of software (NIS-elements D, version 5.3 software) with the function ‘scanning wizard’. This provided an image of the pyloric sphincter of the stomach containing the torus pyloricus of all 36 piglets. Of the 36 images, 25 were suitable for further analysis (*n* = 8 for CON; *n* = 9 for OH-f; *n* = 8 for OH-c); the torus pyloricus could not be identified for the other piglets.

After the localization of the torus pyloricus in the pyloric region, the size of the torus pyloricus was determined by measuring its length and depth, and the mucosal and muscular layer of the torus pyloricus were measured in five different locations per piglet. The width and the longitudinal and circular smooth muscles of the ileocecal sphincter were measured as described in Van Hees et al. (2023b). Paraffin-embedded sections of the cecum were double-stained to allow identification of the different mucin subtypes present as mucin droplets within a goblet cell. Firstly, sections were stained by periodic acid-Schiff, which stained neutral mucins magenta. Secondly, sections were stained by Alcian blue at pH 2.5 which stained the (acid) sialomucins turquoise. The mixed mucins would stain purple. Goblet cell (GC) counts, size, and total area covered by GC were measured based on the staining. Moreover, cecum wall mucosa was measured using a computerized imaging system software (CellSens Dimensions, Olympus Nederland B.V., Leiderdorp, The Netherlands). Crypt depth was determined at three locations per slide.

### Analytical procedures

The experimental diets were analyzed for DM (method 930.15), nitrogen (N) (method 990.03) with CP calculated as N × 6.25, crude ash (method 942.05), crude fat (method 920.39), total DF, SDF and IDF (method 991.43), starch (NEN-ISO 15914:2004), NDF (method 2002.04), and TiO_2_ (method 974.02) (AOAC, 2005). Particle size of the diets (ie, crumbles) was determined by wet sieving using an in-house method (MasterLab, Boxmeer, The Netherlands). Digesta were analyzed using the same laboratory methods. The contents from the distal small intestine and the rectum were analyzed for DM and TiO_2_. The content from the distal small intestine was also analyzed for DM, N and crude fat. The content of the rectum was also analyzed for DM and NDF. The contents of the stomach, cecum, and colon were analyzed for DM, pH, lactic acid concentration, VFA, and ammonia-N. The lactic acid and VFA concentrations were analyzed by HPLC on a BioRad Aminex HPX-87H using a 0.005 mol/L sulfuric acid eluent at a flow rate of 0.7 mL/min. Only the VFA with concentrations above the detection level were reported. Ammonia-N (i.e., N as ${\text{NH}}_{4}^{+}$ and NH_3_) was analyzed calorimetrically using the Berthelot reaction as previously described by Van Hees et al. (2019).

### Calculations

Organ weights were expressed as g/kg BW at dissection to account for differences in BW at dissection between piglets. Apparent digestibility of nutrients in the distal small intestine (apparent ileal digestibility [AID]) and rectum (apparent total tract digestibility [ATTD]) were calculated using the following equation Stein et al. (2007): AID or ATTD, % = 100 − [(nutrient_digesta_/nutrient_feed_) × (TiO_2feed_/TiO_digesta_)] × 100, where nutrient_digesta_ and TiO_2digesta_ where the concentrations of each in the digesta and nutrient_feed_ and TiO_2feed_ where the concentrations of each in the feed.

### Statistical analysis

All statistical analyses were performed in SAS studio (SAS Institute). Data were analyzed using individual piglet as the experimental unit. The BW at dissection (full and empty), empty BW as proportion of BW at dissection, organ weights (full and empty), colon length, relative colon length (empty weight/length), ileocecal sphincter, crypt depth in the cecum, pH and DM in digesta, and nutrient digestibility in the small intestine and rectum were analyzed with PROC MIXED using the following model:


$$Y_{\text{ijkl}}=µ+{\;S}_{i}+{\;T}_{j}+{\;P}_{k}+\varepsilon_{\text{ijkl}}$$

Y_ijkl_ is the dependent variable, µ is the overall mean, S_i_ is the fixed effect of sex, T_j_ is the fixed effect of treatment, P_k_ is the random effect of pen, and ε_ijkl_ is the error. Lactic acid, VFA, and ammonia-N were analyzed by PROC GLIMMIX with sex and treatment as fixed effects, pen as random effect, and a gamma distribution and log link function. The measurements on the stomach pyloric sphincter were analyzed with PROC MIXED using the following model:


$$Y_{\text{ij}}=µ+{\;T}_{i}+{\;P}_{j}+\varepsilon_{\text{ij}}$$

Y_ij_ is the dependent variable, µ is the overall mean, T_i_ is the fixed effect of treatment, P_j_ is the random effect of pen, and ε_ij_ is the error. We considered *P*-values ≤ 0.05 as significant and *P*-values between > 0.05 and ≤ 0.10 as trends. Means were separated using the Tukey-Kramer post-hoc test.

## Results

### General observations

The OH-f diet had a similar particle size distribution compared to the CON diet while OH-c had a higher fraction of particles >1.00 mm (on average 4.7 vs 8.9%, respectively; Table 1). Both the OH-f and OH-c diets had a higher total DF caused by a higher IDF compared to the CON diet (Table 1). Thus, diets were suitable to determine both the effect of IDF as well as the effect of particle size of the IDF fiber.

### Gastrointestinal morphometry

The BW at dissection and empty BW at dissection did not differ between the experimental diets (*P *> 0.050; Table 2). The empty BW as proportion of the BW at dissection was 4% higher (*P *< 0.001) for the CON diet compared to the OH-f and OH-c diets (Table 2). The empty stomach weight was highest for the OH-c diet (9.65 g/kg BW), followed by the OH-f diet (8.79 g/kg BW), and subsequently by the CON diet (7.32 g/kg BW; *P *< 0.001; Table 2). The stomach content weight was higher (*P *= 0.026) for OH-c (36.09 g/kg BW) than for CON (22.43 g/kg BW) with OH-f in between and not different from the other two diets (32.50 g/kg BW). The cecum content weight (+30%; *P *= 0.029), empty colon weight (+9%; *P *= 0.025), and colon content weight (+28%; *P *= 0.025) were higher for the OH-f and OH-c diets compared to the CON diet (Table 2). The relative colon weight (in g/cm) was higher (*P *= 0.040) for the OH-c diet compared to the CON diet with the OH-f diet in between and not different (Table 2).

**Table 2.** Organ weight measurements

Item	CON	OH-f	OH-c	Pooled SEM	P-value
Bodyweight (BW) at dissection, kg	9.9	10.2	10.1	5.50	0.790
Empty BW, kg^1^	7.7	7.6	7.5	0.84	0.885
Empty BW as proportion of BW at dissection	0.77^a^	0.74^b^	0.74^b^	0.006	<0.001
Empty stomach weight, g/kg BW	7.32^c^	8.79^b^	9.65^a^	0.218	< 0.001
Stomach content weight, g/kg BW	22.43^b^	32.50^ab^	36.09^a^	3.506	0.026
Empty cecum weight, g/kg BW	2.70	2.65	2.78	0.088	0.570
Cecum content weight, g/kg BW	4.90^b^	6.17^a^	6.57^a^	0.633	0.029
Empty colon weight, g/kg BW	10.34^b^	11.31^a^	11.13^a^	0.326	0.025
Colon content weight, g/kg BW	18.02^b^	23.50^a^	22.74^a^	1.810	0.025
Colon length, cm	171	181	166	10.4	0.110
Colon weight/length, g/cm	0.60^b^	0.63^ab^	0.68^a^	0.043	0.040

Organ weights (LSMeans and pooled SEM) in nursery piglets that received one of three experimental diets: (1) CON = low fiber control diet, (2) OH-f consisting of CON + 15% finely ground oat hulls, or (3) OH-c consisting of CON+ 15% coarsely ground oat hulls. Data are from piglets (*n* = 12 per treatment) subjected to post-mortem evaluation on day 13 after weaning.

a–c Values within a row with different superscripts differed significantly at *P *< 0.050.

1 Empty BW measured after removal of the abdominal organs.

### Histological measurements of the stomach and cecum

There was no difference between experimental diets regarding the measurements of the stomach pyloric sphincter, ie, length, depth, mucosa depth, and muscular layer depth (*P *> 0.050; Table 3). The ileocecal sphincter width was 14% greater for the OH-c diet compared to the CON and OH-f diets (*P *= 0.005; Table 3). The ileocecal sphincter total muscle width tended (*P *= 0.064) to be 25% greater for the OH-C diet compared to the CON diet with the OH-f diet being in between and not different. There were no differences between experimental diets for crypt depth (*P *= 0.683) and the average size of the GC (*P *> 0.050), and only numerical differences for the area covered by total GC (*P *= 0.135) and total GC count (*P *= 0.118; Table 3).

**Table 3.** Histological measurements of the stomach pyloric sphincter, ileocecal sphincter, and cecum crypt depth and goblet cells (GC)

Item	CON	OH-f	OH-c	Pooled SEM	*P*-value
**Stomach pyloric sphincter**					
N	8	9	8		
Length, mm	5.80	5.74	5.32	0.560	0.811
Depth, mm	2.91	2.50	2.71	0.254	0.527
Mucosa depth, µm	632	686	674	47.9	0.710
Muscular layer depth, µm	146	130	130	13.2	0.651
**Ileocecal sphincter**					
N	12	12	12		
Ileocecal sphincter width^1^, mm	8.38^b^	8.77^b^	9.74^a^	0.268	0.005
Ileocecal sphincter longitudinal muscle^2^, µm	209	223	250	19.8	0.322
Ileocecal sphincter total muscle width^3^, µm	951^y^	973^xy^	1184^x^	75.5	0.064
**Cecum**					
Crypt depth, µm	428	440	426	12.4	0.683
**Area covered by GC^4^ in cecum, %**					
Total GC	6.51	8.26	10.59	1.387	0.135
Acid GC	2.27	2.65	3.36	0.463	0.259
Neutral GC	0.90	1.29	1.61	0.425	0.508
Mixed GC	3.35	4.31	5.62	0.874	0.204
**GC count in cecum**					
Total GC	824	1029	1107	97.0	0.118
Acid GC	267	328	358	37.5	0.233
Neutral GC	141	173	186	31.7	0.588
Mixed GC	415	528	563	52.2	0.129
**Average size^4^ in cecum, µm^2^**					
Total GC	53.1	56.5	61.1	5.58	0.612
Acid GC^4^	56.1	56.5	59.4	6.16	0.920
Neutral GC^4^	37.1	41.0	52.3	7.11	0.313
Mixed GC^4^	51.3	57.1	62.4	6.89	0.533

Data are presented as LSMeans and pooled SEM in nursery piglets that received one of three experimental diets: (1) CON = low fiber control diet, (2) OH-f consisting of CON + 15% finely ground oat hulls, or (3) OH-c consisting of CON+ 15% coarsely ground oat hulls. Data are from piglets subjected to post-mortem evaluation on day 13 after weaning.

a, b Values within a row with different superscripts differed significantly at *P *< 0.050.

x, y Values within a row with different superscripts show a trend at *P *< 0.010.

1 *n* = 8 for CON, *n* = 10 for OH-f and *n* = 10 for OH-c.

2 *n* = 12 for CON, *n* = 10 for OH-f and *n* = 11 for OH-c.

3 *n* = 12 for CON, *n* = 10 for OH-f and *n* = 12 for OH-c.

4 *n* = 12 for CON, *n* = 12 for OH-f and *n* = 11 for OH-c.

### Stomach digesta metabolites

The pH in the fundic area of the stomach was higher for the OH-c diet compared to the CON and OH-f diets (*P *= 0.004) while the pH in the pyloric area was not different (*P *= 0.904; Table 4). This resulted in a greater pH gradient for the OH-c diet compared to the CON and OH-f diets (*P *< 0.001; Table 4). The DM in the proximal region of the stomach was highest for the OH-c diet, followed by the OH-f diet, and subsequently the CON diet (*P *< 0.001) while there was no difference in DM in the distal region (*P *= 0.183). Thus, also for DM the gradient between the proximal and distal region was highest (*P *= 0.025) for the OH-c diet compared to the CON diet with the OH-f diet in between and not different (Table 4). The gastric lactic acid concentration was 380% greater for the OH-f and OH-c diets compared to the CON diet (*P *= 0.001; Table 4). The total VFA concentration in the stomach was greatest for the OH-c diet followed by the OH-f diet and subsequently the CON diets (*P *< 0.001) which was mainly caused by a greater acetic acid (*P *< 0.001; Table 4) The ammonia-N concentration in the stomach was 41% greater in the CON and OH-f diets compared to the OH-c diet (*P *= 0.018; Table 4).

**Table 4.** Stomach digesta metabolites

Item	CON	OH-f	OH-c	Pooled SEM	*P*-value
pH, fundic area	3.40^b^	3.62^b^	4.33^a^	0.189	0.004
pH, pyloric area	3.52	3.40	3.39	0.237	0.904
pH gradient	−0.12^b^	0.23^b^	0.93^a^	0.175	< 0.001
Dry matter, proximal region, %	14.4^c^	18.9^b^	25.8^a^	1.38	< 0.001
Dry matter, distal region, %	23.8	23.2	28.1	2.08	0.183
Dry matter gradient	−9.4^b^	−4.3^ab^	−2.3^a^	1.80	0.025
Lactic acid, mmol/L	6.3^b^	24.4^a^	23.4^a^	8.24	0.001
Acetic acid, mmol/L	21.6^c^	66.8^b^	115.5^a^	5.20	< 0.001
Succinic acid^1^, mmol/L	2.1^b^	4.4^a^	3.3^a^	0.57	0.001
Total VFA^3^, mmol/L	23.8^c^	77.8^b^	118.6^a^	6.66	< 0.001
Ammonia-N, mmol/L	96.8^a^	99.2^a^	69.3^b^	8.25	0.018

Stomach digesta metabolites concentration (mmol/L unless stated otherwise; LSMeans and pooled SEM), dry matter (DM) and pH of gastrointestinal contents in nursery piglets (*n* = 12 per treatment) that received one of three experimental diets: (1) CON = low fiber control diet, (2) OH-f consisting of CON + 15% finely ground oat hulls, or (3) OH-c consisting of CON+ 15% coarsely ground oat hulls. Data are from piglets subjected to post-mortem evaluation on day 13 after weaning.

a, b Values within a row with different superscripts differed significantly at *P *< 0.050.

x, y Values within a row with different superscripts show a trend at *P *< 0.010.

### Volatile fatty acid concentration in cecum and colon digesta

In the cecum, we observed a higher DM% for the OH diets compared to the CON diet (*P *= 0.033; Table 5), while no differences in pH were observed (*P *= 0.712). Meanwhile the acetic acid concentration was highest (*P *= 0.003) in the OH-c diet compared to both the CON and OH-f diets, which were not different from each other (Table 5). A numerical difference was observed for the concentration of butyric acid (*P *= 0.134), while for propionic acid, there was a tendency (*P *= 0.063) for a higher concentration in the OH-c compared to the OH-f diet (Table 5). These observations resulted in a higher concentration of total VFA in the OH-c diets (*P *= 0.029) compared to both the CON and OH-f diets, which were not different from each other (Table 5). Finally, for the cecum, both OH diets had a reduced ammonia-N concentration compared to the CON diet (*P *= 0.006; Table 5).

**Table 5.** Volatile fatty acid (VFA) concentration in the cecum and colon

Item	CON	OH-f	OH-c	Pooled SEM	*P*-value
**Cecum**					
pH	5.92	5.81	5.86	0.09	0.712
Dry matter, %	14.6^b^	19.0^a^	19.0^a^	1.3	0.033
Acetic acid, mmol/L	149.7^b^	168.1^b^	211.1^a^	11.72	0.003
Propionic acid, mmol/L	50.4^xy^	42.7^y^	54.2^x^	3.45	0.063
Butyric acid^1^, mmol/L	25.9	18.3	22.1	2.63	0.134
Total VFA^2^, mmol/L	229.7^b^	231.5^b^	288.6^a^	16.24	0.029
Ammonia-N, mmol/L	43.5^a^	28.7^b^	29.5^b^	3.18	0.006
**Colon**					
pH	6.32	6.21	6.31	0.089	0.639
Dry matter, %	23.4	23.5	25.6	0.92	0.135
Acetic acid^3^, mmol/L	137.8^b^	200.6^a^	180.0^a^	15.24	< 0.001
Propionic acid^3^, mmol/L	36.6^x^	35.9^xy^	26.5^y^	3.55	0.070
Butyric acid^3^, mmol/L	17.2^a^	15.4^a^	10.8^b^	2.72	0.021
Total VFA^2^ ^,3^, mmol/L	195.6^c^	256.3^a^	232.7^ab^	19.89	0.049
Ammonia-N, mmol/L	40.4^a^	24.6^b^	20.2^b^	4.36	< 0.001

The VFA concentration (mmol/L unless stated otherwise; LSMeans and pooled SEM), dry matter and pH of gastrointestinal contents in nursery piglets that received one of three experimental diets: (1) CON = low fiber control diet, (2) OH-f consisting of CON + 15% finely ground oat hulls, or (3) OH-c consisting of CON+ 15% coarsely ground oat hulls. Data are from piglets subjected to post-mortem evaluation on day 13 after weaning.

a, b Values within a row with different superscripts differed significantly at *P *< 0.050.

x, y Values within a row with different superscripts show a trend at *P *< 0.010.

1 *n* = 11 for CON, *n* = 11 for OH-f, and *n* = 11 for OH-c.

2 Sum of acetic acid, propionic acid, butyric acid, valeric acid and succinic acid.

3 *n* = 12 for CON, *n* = 11 for OH-f, and *n* = 12 for OH-c.

In the colon, we observed no treatment differences in DM% and pH measurements (*P *> 0.050; Table 5). The OH diets had a 31%–38% higher acetic acid concentration compared to the CON diet (*P *< 0.001), while for butyric acid, the CON and OH-f diet were both similar and higher (51%) than the OH-c diet (*P *= 0.021; Table 5). The propionic acid concentration tended to be lower in the OH-c diet compared to the CON diet with the OH-f diet in between and not different (*P *= 0.070; Table 5). As a result, the total VFA concentration was greatest for the OH-f diet, followed by the OH-c diet and the CON diet being lowest (*P *= 0.049; Table 5). Similar to the cecum, the ammonia-N concentration was lower in OH diets compared to the CON diet (*P *< 0.001; Table 5).

### Apparent ileal and total tract digestibility

There was a tendency for higher AID of DM in the CON diet compared to the OH-c diet while the OH-f was intermediate (*P *= 0.078; Table 6). The AID of CP (*P *= 0.953) and crude fat (*P *= 0.257) were not different between treatments (Table 6). The ATTD of DM was not different among the treatments (*P *= 0.618), while the ATTD of NDF, was higher in the OH diets compared to the CON diet (*P *< 0.001; Table 6).

**Table 6.** Apparent ileal (AID) and total tract (ATTD) digestibility

Item	CON	OH-f	OH-c	Pooled SEM	P-value
**AID^1^**					
Dry matter	96.2^x^	95.5^xy^	95.3^y^	0.30	0.078
Crude protein	73.5	73.6	74.0	1.08	0.953
Crude fat	86.9	88.1	88.6	0.73	0.257
**ATTD**					
Dry matter	92.3	92.2	91.8	0.43	0.618
NDF^2^	9.4^b^	48.1^a^	51.1^a^	3.49	< 0.001

Data for the AID and ATTD (%, LSMeans and pooled SEM) in nursery piglets that received one of three experimental diets: (1) CON = low fiber control diet, (2) OH-f consisting of CON + 15% finely ground oat hulls, or (3) OH-c consisting of CON+ 15% coarsely ground oat hulls. Data are from piglets subjected to post-mortem evaluation on day 13 after weaning.

a, b Values within a row with different superscripts differed significantly at *P *< 0.050.

x, y Values within a row with different superscripts show a trend at *P *< 0.010.

1 *n* = 12 for CON, *n* = 12 for OH-f, and *n* = 11 for OH-c.

NDF^2^; neutral detergent fiber.

## Discussion

The goal of the present study was to test the effects of the inclusion of OH, either finely or coarsely ground, in diet for weanling piglets, to ascertain its efficacy in stimulating GIT development and function mainly through the primary action of IDF and secondary action of particle size.

The impact of DF, in general, on GIT development and function is well reported in piglets (Lindberg, 2014; Priester et al., 2020; Wellington et al., 2020), however, data specific on piglets early post-wean is scarce. This is largely because, typically, weanling piglet diets are formulated with highly digestible and absorbable nutrients to avoid issues with poor nutrient intake and protein fermentation metabolites (Wensley et al., 2021). However, recent studies suggested that weanling piglets could also benefit from the inclusion of fiber in their diets, especially SDF and IDF fractions (Van Hees et al., 2019; Chen et al., 2020; Zhu et al., 2024). Therefore, using OH, as a source of IDF in the present study concurs with other studies in weanling piglets, that fed OH to weanling piglets to improve GIT development either during the pre-weaning or the post-weaning period (Mateos et al., 2006; Van Hees et al., 2023b).

The focus of the current study was to alter GIT development by means of OH. The period of measuring growth performance was rather short (12 d), and the study was not powered to detect growth performance differences in that short period. Another part of this study, with different animals but of the same weaning batch, had sufficient power but also here no differences in growth performance were observed (Van Hees, 2022).

In contrast to the current study, weanling piglets fed diets in which 150 or 200 g/kg soybean meal was substituted with wheat distillers dried grains, showed a reduction in growth performance, even though the diets were isoenergetic and isonitrogenous (Avelar et al., 2010). In a review by Flis et al. (2017) on fiber effects on weaned piglets, the authors suggested that the effect of fiber source and inclusion level of DF to improve growth performance and GIT development in weaned piglets are independent from each other. For example, an inclusion of 4%–8% coarse wheat bran was recommended to promote GIT development and growth, while an inclusion of 2% OH was recommended to achieve the same effect. These observations are indicative that fiber source and inclusion level can influence the outcomes of a study. Moreover, within a fiber source, the particle size and the secondary processing of the ingredient mix does not seem to affect growth performance. For example, Mateos et al. (2006) used cooked and expanded OH in the diet that was fed as mash, Kim et al. (2008) hammer-milled the OH on a 4.5 mm screen, and OH beater-cross milled on a 3 and subsequently 1.5 mm screen or OH milled on a 3.0 mm screen were used in the current study. These observations are indicative that perhaps the level and particle size of the fiber source may not exert as much influence compared to the fiber fraction itself depending on the response variable of interest (e.g., growth vs GIT development). Since each fiber source may be composed differently, it is more relevant to compare the fiber fractions (i.e., total DF, SDF, and IDF) and their influence on performance and GIT development. However, in the current study, despite small differences in the particle size distribution (4.5 vs. 8.9% above 1 mm, between the OH-f vs. OH-c) we did find some effects specifically related to the particle size of the IDF fraction.

We hypothesized that next to a higher IDF level, the coarseness of the IDF would bring an additional stimulation of GIT development and function. Therefore, the OH were either finely or coarsely ground and subsequently included in an ingredient mix that was pelleted and subsequently crumbled. Typically, secondary processing reduces particle size (Vukmirović et al., 2017; Lyu et al., 2020) but in the current diets the OH-c diet had two times more particles >1000 µm compared to the CON and OH-f diets. Coarser particles have mainly been studied in regard to effects on GIT health (Molist et al., 2010; Molist et al., 2012; Patience and Ramirez, 2022) and development (Cappai et al., 2015; Hulshof et al., 2024). In the current study, although there was an IDF effect, the empty stomach weight was further increased for the OH-c diet, which matched with observations on diets containing coarse cereals (Hulshof et al., 2024). Moreover, the ileocecal sphincter width was larger in the OH-c fed pigs, indicative of an improved development of that valve, which concurs with Cappai et al. (2015).

The gastric pH in the fundic area was highest for OH-c resulting in the greatest pH gradient in the stomach. This effect was not seen for CON and OH-f and, thus, appeared to be the result of the larger particles (Mößeler et al., 2012). In contrast, Bornhorst et al. (2013) found that both brown and white rice resulted in a pH gradient in growing pigs and, thus, that the effect depended on IDF content. The observed effects on pH can be mediated by effects on retention time of the fine solids and fibrous particles which was found to be greater in finishing pigs fed a diet containing coarse straw compared to fine straw (Lannuzel et al., 2024). Retention time was not determined in the current study but the effects on DM of the gastric content (proximal and gradient) indicated that retention time was greatest for OH-c, followed by OH-f, and subsequently CON. A greater pH gradient is typically considered as beneficial for young pigs since that will support pre-digestion in the stomach and prevent the formation of stomach ulcers (Huting et al., 2021). Ulcers were not observed in the current study, but 83% of CON and 42% of OH-f pigs showed signs of mild keratinization in the *pars non-glandularis* area of the stomach, while no mild keratinization was observed in the OH-c fed pigs. The soluble and insoluble fractions of fiber are noted as the fractions that exert different physicochemical properties in the GIT (Lannuzel et al., 2024). As such, an increase in the DF content for weanling piglets could either be beneficial or detrimental based on the composition and physicochemical properties of the fiber source.

In the present study, the OH diets contained high concentrations of IDF (CON; 10.2%, OH-f; 17.8%, and OH-c; 19.5%), while the differences in SDF between the CON and OH diets were only minimal (CON; 1.8%, OH-f; 2.1%, and OH-c; 2.3%). Therefore, the main differences observed in the present study on the measured parameters can be largely attributed to the dietary concentration of IDF. Studies have shown that the inclusion of high DF (IDF or SDF) leads to increased VFA production, particularly butyric and acetic acid (Molist et al., 2009). The VFA are noted to confer positive effects on intestinal health and performance in piglets (Tonel et al., 2010; Bedford and Gong, 2018; Lauridsen, 2020). For example, a high butyrate concentration is reported to increase intestinal cell proliferation and acts as fuel for enterocytes (Kien et al., 2007) in such a way that increased butyrate production is improving intestinal health and performance in piglets (Huang et al., 2015; Zhao et al., 2018).

In the current study, the total VFA concentrations in the stomach, cecum, and colon were increased with the OH diets relative to the control diet. This concurs with many reports on the effect of high DF on total VFA production (Jha and Berrocoso, 2015; Zhao et al., 2019). As previously mentioned, butyrate is one of the most important VFA for cell proliferation and general intestinal health, as such the concentration of butyrate is important. In the present study, butyrate concentrations were undetectable in the stomach, and no differences were observed in the cecum. In the colon, contrary to expectations, butyrate concentrations were lower in the OH-c compared to the OH-f and CON which were not different, suggesting that perhaps the high IDF and coarseness of the OH negatively affected the butyrate producing microbial community which may have impacted fermentation and metabolite production.

Previous studies have indicated that the type of DF fraction (IDF or SDF) can affect microbial diversity, fermentation and metabolite production by shifting the butyrate producing microbial community which will affect total butyrate production (Metzler-Zebeli et al., 2010; Ying et al., 2013; Li et al., 2016). Acetic acid production on the other hand was increased in the OH diets compared to the CON diet, in both the cecum and colon. This observation aligns potentially with a shift in the microbial community occasioned by the OH used in the experimental diets, thereby, increasing fermentation in support of high acetic acid production (Zhang et al., 2018). Also, higher amounts of VFAs are produced from the SDF fraction as compared to the IDF fractions (Urriola and Stein, 2010; Jha and Berrocoso, 2015; Zhao et al., 2020), as such, in the present study, where high OH (higher IDF) diets were fed, it would be expected that the butyrate production will be lower especially in the cecum, than colon. Taken together, compared to the CON diet, the production of high amounts of VFAs, in OH diets, provides a much better developmental support of the gut, hence presents a better opportunity for enhanced piglet growth during the immediate post-weaning period.

Further, we observed a higher empty colon weight for the OH diets compared to the CON diet, also the relative colon weight (weight/length) was higher indicating improved development, an observation that concurs with previous reports (Agyekum et al., 2012; Hong et al., 2021; Yao et al., 2025). The content weights of the stomach, cecum, and colon were higher for the OH diets compared to the CON diet resulting in a lower empty BW as proportion of BW at dissection. The contents of the stomach and cecum were also drier (i.e., higher DM%). Regarding the stomach, this may indicate a higher retention time of solids in the stomach and, thereby, a more controlled flow of nutrients into the small intestine, with subsequent better nutrient digestion and more time for fermentation. The effect of IDF on mean retention time depends on the location in the GIT and fraction, that is, liquid, solid, fibrous (Wilfart et al., 2007; Lannuzel et al., 2024). Inclusion of 15% OH either fine or coarse numerically increased the total GC count and area covered. Goblet cell size and numbers are important in mucus secretion, which is important in protecting the intestinal lining (Wellington et al., 2020).

Taken together, diluting a nutrient-dense diet by inclusion of OH (15%) did not result in a reduced growth performance of piglets in the immediate post-weaning period, perhaps due to a higher protein to NE ratio in the OH based diets. This may be due to a higher NE efficiency in piglets fed the OH diets as also reported for high IDF diets by Yao et al. (2025). The higher NE efficiency in the OH fed piglets may be due to a more developed and functional GIT (i.e., increased goblet cell counts, increased organs weights and increased total VFA production), even more pronounced when the OH were in a coarse form. The observed increase in fermentation activity in the large intestine (cecum and colon) with the OH diets, resulted in higher VFA concentrations, which is known to confer additional benefits to the intestinal epithelium but also leads to an increase in ME to support metabolic activities. Assessing the impact of the OH on relative organ weights and GC suggested improved development and function compared to piglets fed the CON diet. As such, the concept of diluting a weanling piglet diet with OH appears to confer benefits on GIT development while not negatively affecting growth performance of the piglets, as demonstrated in the present study.

## Conclusion

Including oat hulls as a source of IDF in weanling piglet diet was shown to enhance intestinal morphological and functional development, especially when the OH was in a coarsely ground form. These positive effects of oat hulls observed in the present study, may offer opportunities to improve piglets’ resilience to post-weaning diarrhea and other gut health challenges in the post-weaning phase.

## Abbreviations:

AID: apparent ileal digestibility
ADFI: average daily feed intake
ADG: average daily gain
ATTD: apparent total tract digestibility
BW: bodyweight
CON: control diet
CP: crude protein
DF: dietary fiber
DM: dry matter
; EFS: electronic feeding station
GC: goblet cell
GE: gross energy
GIT: gastrointestinal tract
IDF: insoluble dietary fiber
NDF: neutral detergent fiber
OH: oat hulls
OH-c: oat hulls coarse diet
OH-f: oat hulls fine diet
SDF: soluble dietary fiber
SD: standard deviation
VFA: volatile fatty acid
